# Impact of Oral Health Educational Interventions on Oral Hygiene Status of Children with Hearing Loss: A Randomized Controlled Trial

**DOI:** 10.1155/2021/5185613

**Published:** 2021-11-17

**Authors:** Maria Moin, Sohail Saadat, Sara Rafique, Afsheen Maqsood, Abhishek Lal, Fahim Vohra, Mohammad Khursheed Alam, Naseer Ahmed

**Affiliations:** ^1^Department of Community Dentistry, Bahria University Medical and Dental College, Karachi 75530, Pakistan; ^2^Department of Community Dentistry, Doctor Ishrat ul Ebad Khan Institute of Oral Health Sciences, Karachi 7422, Pakistan; ^3^Department of Physiology, Jinnah Medical and Dental College, Karachi 74800, Pakistan; ^4^Department of Oral Pathology, Bahria University Medical and Dental College, Karachi 75530, Pakistan; ^5^Department of Prosthodontics, Altamash Institute of Dental Medicine, Karachi 75500, Pakistan; ^6^Department of Prosthetic Dental Science, College of Dentistry, King Saud University, Riyadh, Saudi Arabia; ^7^Department of Preventive Dentistry, College of Dentistry, Jouf University, Sakaka, Al Jouf 72345, Saudi Arabia; ^8^Prosthodontics Unit, School of Dental Sciences, Health Campus, Universiti Sains Malaysia, 16150 Kubang Kerian, Kota Bharu, Kelantan, Malaysia

## Abstract

**Introduction:**

Oral health is considered as one of the essential components of the overall health of every individual. Maintaining oral health is a gradual process that requires commitment. Children who require special care such as hearing impairment experience difficulty in maintaining oral health primarily due to communication difficulties. This study is aimed at using different interventions to evaluate the improvement of oral hygiene in hearing impaired children.

**Materials and Methods:**

Fifty-nine children were recruited in this study that were allocated randomly into each group with twenty children as follows: group 1: pictorial, group 2: video, and group 3: control. Mean plaque and gingival scores were noted before and after the use of different interventions. Oral hygiene was categorized as “excellent,” “good,” and “fair.” Gingival health was categorized as “healthy,” “mild gingivitis,” and “moderate gingivitis.”

**Results:**

Thirty-four children (57.6%) were from 12-13 years of age bracket, and 25 (42.4%) belonged to 14-16 years of age. Regarding gender, there were 37 (62.7%) males and 22 (37.3%) females. About comparison of mean gingival and plaque scores before and after interventions in each group, a significant difference was found in group 1 (*p* < 0.001) and group 2 (*p* < 0.001), as compared to group 3 where the difference in scores was not significant (*p* > 0.05).

**Conclusion:**

Maintaining oral health requires the compliance of individuals to perform different methods of preventive dentistry, such as tooth brushing and use of dental floss. The use of different oral hygiene educational interventions such as pictorial and video methods have been proven and useful for hearing impaired children in improving oral health.

## 1. Introduction

Oral health is one of the most fundamental elements for maintaining the general physical health and well-being of every individual. A healthy mouth performs many of the vital functions such as eating, speaking, and participating in facial expressions effortlessly. The most visible part of the mouth in terms of esthetics is the teeth. Teeth are the first thing that people notice when it comes to socializing. Healthy and decay-free teeth are able to perform many of their functions such as mastication and speech, along with providing self-confidence to individuals, as esthetics is equally important to many individuals [1].

The children are more prone to develop oral health problems primarily due to the lack of attention being given by them, as compared to adults who are well mature to understand the importance of healthy oral hygiene. Furthermore, children who require special care in order to maintain their oral hygiene are even more vulnerable to develop oral health difficulties because of the illness they suffer from [2].

Hearing disability is one of the major disabilities that is faced by children globally [3]. According to one study, there are 23 to 25 thousand children aged between 0 and 15 years in the United Kingdom who have a hearing disability [3]. Children who are born with deafness as a disability or those who acquire it at any period of time undergo a series of traumatic episodes which makes taking care of oral hygiene an insignificant aspect of life [4]. A child whose hearing has been impaired due to any reason endures a difficult time socializing with people, learning, communicating, and cognitively lacks behind normal children [5].

Educating children as well as adults has been a vital part of preventive dentistry that has evolved over a period of time in order to improve the oral hygiene of individuals [5]. Preventive dentistry begins at the level of primary school that includes a visual presentation of brushing and the importance of oral health through various educational videos [6]. Furthermore, children are also encouraged to participate in various school-based activities which are part of their school curriculum such as poster competitions, essay writing, and oral presentation on various aspects of the importance of oral health [7]. However, such strategies for children with impaired hearing have not been very useful. Many different strategies have been explored and put to work that is described in various studies such as using playful learning interventions for children [8].

Normally, many oral diseases such as dental caries, gingivitis, periodontitis, and even dental fracture can easily be prevented in children, but due to hearing disability, these children are more prone to suffer from such dental diseases [9]. Numerous methods can be used to maintain adequate oral hygiene such as toothbrushes, dental floss, chewing gums, interdental toothbrushes, mouthwashes, and dentifrices as well [9]. However, for these interventions to be beneficial, manual dexterity is the main hindering factor for the children with impaired hearing as communicating with them to explain the importance of each of these interventions is a challenging task [10]. According to American Dental Association (ADA) and British Dental Association (BDA), dental floss along with toothbrushes has been recommended to be daily used by all individuals [11].

In this study, we aimed to compare the efficacy of pictorial and video demonstration methods as oral health education interventions, to evaluate changes in oral hygiene of children who had a hearing impairment.

## 2. Materials and Methods

### 2.1. Study Setting and Sample Size Calculation

This randomized controlled trial study was carried out from October 2019 to January 2020 in 3 different schools of Karachi, Pakistan, that were dedicated to the education of children who require special care. To calculate the sample size, OpenEpi software was used keeping the confidence interval at 95% and power of test 80%. The sample size was calculated to be 60 participants (20 in each of the 3 groups) [12].

### 2.2. Ethical Consideration and Participant Recruitment

The ethical approval was granted by the ethical review committee of Altamash Institute of Dental Medicine, Pakistan; number (AIDM/ERC/02/2019/07). The trial was registered under “clinicaltrials.gov” (United States National Library of Medicine). This study is composed in line with CONSORT guidelines for reporting clinical trials [13]. The protocol of the study was explained to parents of all the children included in this study, as well as the administration of the school. After explaining the protocols of this study, written and verbal consent was taken. Hearing-impaired children who were aged between 12 and 16 years of age who showed a willingness to participate in this study were recruited. Furthermore, children with no systemic illness, with plaque adhering to the free gingival margins more than one-third and more than two-thirds of the tooth surface. The children with mild, moderate, and severe gingivitis and those being able to comply with the protocols of this study were included in this study. Those children who required antibiotics prior to the start of the study and with extensive dental calculus were excluded from this study.

### 2.3. Sampling Technique

In this study, a purposive sampling technique was used; there were twelve schools for disabled children in Karachi city. Out of these twelve, three schools IDAREU, Deaf Reach, and JS Academy for deaf agreed to participate in the study that is why these three were selected. The Deaf Reach School had a total of one-hundred and eighty-six hearing impaired children; out of them, sixty were between 12 and 16 years of age; IDAREU had three hundred and fifty hearing impaired children; out of them, sixty were 12-16 years of age; and JS Academy had one-hundred and fifty hearing impaired children; out of them, 51 were 12-16 years of age.

### 2.4. Grouping and Randomization of the Participants

**Figure 1:**
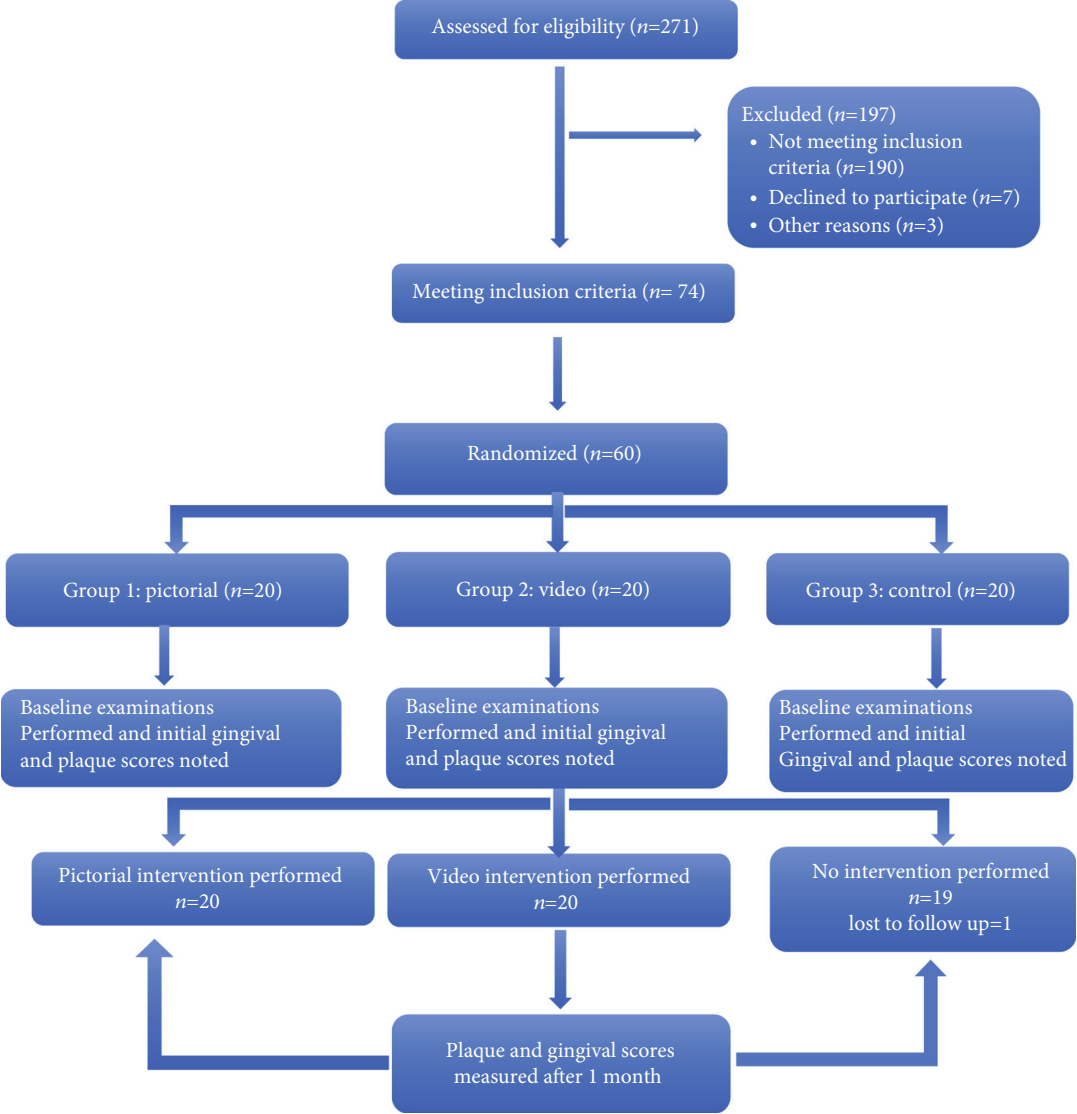
CONSORT flow diagram of the study.

The eligibility criteria were applied to all the two-hundred and seventy-one hearing impaired children with 12-16 years of age. Seventy-four children were found eligible for this trial. Out of these seventy-four children, through randomization, sixty children were selected using a `random number table by creating a numbered list. Out of the sixty children selected, randomly, each of the twenty children were allocated to three different oral health educational interventions such as group 1 (pictorial), group 2 (video), and group 3 (control) as shown in Figure 1. Participants in group 1 were assigned a pictorial method of oral hygiene intervention. They were shown various pictures of ways to maintain oral hygiene such as brushing techniques, use of mouthwash, and dental floss. Participants in group 2 were assigned a video playing method of oral hygiene intervention, and participants in group 3 did not receive any intervention during the study.

### 2.5. Application of Oral Hygiene Educational Intervention

To assess the current oral hygiene of the participants in each group, baseline examinations were performed using sterilized dental instruments on a wooden chair using natural sunlight. A proforma was designed that included personal details of the participants along with oral examination tools. The current plaque and gingival scores of the participants were recorded using plaque index (PI) [14] and gingival index (GI) [15] with the Michigan O probe that is recommended by the World Health Organization (WHO). The plaque and gingival scores were documented on the mesial, distal, and middle surface of the buccal and lingual surface of six index teeth which are 16, 12, 24, 44, 32, and 36, respectively. A total of six measurements were taken, and their average score was then calculated. The clinical oral examination of each of the participants was performed by two examiners (M.M.) and (S.R.), with the examination of all index teeth done, and an average score was calculated for each participant in this study. The oral hygiene of each individual was categorized by oral hygiene score as follows: 0 = excellent, 0.1-0.99 = good, 1.0-0.99 = fair, and 2.0-3.0 = poor. The gingival status of each individual was categorized by gingival score as follows: 0 = healthy gingiva, 0.1-1.0 = mild gingivitis, 1.1-2.0 = moderate gingivitis, and 2.1-3.0 = severe gingivitis.

After the clinical oral examination of the children was performed, they were randomly assigned to each of the three groups. All of the children in each of the 3 groups received a toothbrush as a standard. For children in group 1, they received tooth brushing instructions by formulating laminated cards that presented each step of performing tooth brushing as they were asked to take the cards with them home and mimic the steps shown in the pictures while they brush their teeth. For group 2, a video demonstration method of oral hygiene intervention was used where the children were shown a video of 10 minutes where a cartoon character presented ways of tooth brushing and showed the importance of having good oral hygiene, and the children were then asked to see the video and mimic the steps shown to them while they brush their teeth. In both pictorial and video demonstration methods, the recommended tooth brushing technique was “horizontal scrub technique” and the duration and frequency of the toothbrush were set at twice daily for two minutes. For group 3, these children only received a toothbrush, and they were not provided with any sort of brushing technique instructions.

### 2.6. Motivation Sessions

In order to keep the children motivated and encouraged to follow their assigned intervention, motivational sessions were kept for them by making them repeat either the pictorial or video method depending on their groups. Two motivational sessions were kept, with a gap of 12-14 days between each of the two sessions for one month. However, children in group 3 did not receive any motivational sessions. The motivation sessions were performed in the first, second, and third month of data collection. The sessions were performed in the three different schools.

### 2.7. Follow-Up Examination

After a period of 1 month, the plaque and gingival scores in all of the participants in the 3 groups were recorded to assess the impact of oral hygiene education interventions, and these scores were then compared to the baseline scores that were recorded at the beginning of the study. The changes in plaque index and gingival index before (preintervention) and after (postintervention) the oral hygiene education were compared in each of the 3 groups.

### 2.8. Statistical Analysis

For the statistical analysis, Statistical Package for the Social Sciences software (IBM, SPSS Statistics, version 25, Chicago, Illinois, United States) was used. The mean values and standard deviation were calculated for plaque and gingival scores along with descriptive analysis such as frequencies and percentages of the given data. In each of the groups, preintervention and postintervention scores were recorded. A one-way ANOVA test was used to see any statistically significant difference between preintervention and postintervention scores within and between each group. A *p* value of ≤0.05 was considered to be as statistically significant.

## 3. Results

In this study, a total of sixty children were recruited; out of which, fifty-nine successfully completed the study due to one child being lost in follow-up after 1 month, due to unknown reasons. Of the 59 children, there were 37 males (62.7%) and 22 females (37.3%). Regarding age, 34 (57.6%) belonged to 12-13 years of age; 25 (42.4%) belong to 14-16 years of age. In group 1 (pictorial), there were 20 hearing impaired children; in group 2 (video) there were 20 children, and 19 children in group 3 (control). In both groups 1 and 2, there were 12 children aged between 12 and 13 years and 8 children between 14 and 16 years of age. For group 3, there were 10 children aged between 12 and 13 years and 9 children between 14 and 16 years of age. About the gender of each group, there were 11 males and 9 females in groups 1 and 2 and 15 males and 4 females in group 3 (as presented in Table 1).

For group 1, the mean plaque score preintervention for group 1 was 1.53 ± 0.20, group 2: 1.48 ± 0.26, and group 3: 1.50 ± 0.23. After the intervention, the mean plaque scores were as follows: group 1: 0.36 ± 0.26, group 2: 0.60 ± 0.40, and group 3: 0.94 ± 0.43. The mean difference in reduction of plaque scores for each group was as follows: group 1: 1.17, group 2: 0.88, and group 3: 0.56. The mean score difference was higher for group 1 (1.17) as compared to group 2 and group 3. A statistically significant difference (*p* < 0.001) was noted between the postintervention levels in the study and control groups, whereas no significant difference (*p* = 0.807) was found in plaque scores at preintervention levels (as presented in Table 2).

For group 1, the mean gingival score preintervention for group 1 was 1.29 ± 0.22, group 2: 1.39 ± 0.30, and group 3: 1.47 ± 0.26. After the intervention, the mean gingival scores were as follows: group 1: 0.29 ± 0.23, group 2: 0.44 ± 0.37, and group 3: 0.80 ± 0.42. The mean difference in reduction of gingival scores for each group was as follows: group 1: 1.00, group 2: 0.95, and group 3: 0.67. The mean score difference was higher for group 1 (1.00) as compared to group 2 and group 3. A statistically significant difference (*p* < 0.001) was found between the gingival scores at postintervention levels (as presented in Table 3).

About the comparison of OHI and GI scores among the 3 group postinterventions, a statistically significant difference was found between group 1 and group 3 (*p* < 0.001). For group 1 and group 2 OHI and GI scores, no statistically significant difference was found (*p* = 0.290). Similarly, a statistically significant difference was found between OHI and GI scores of groups 2 and 3 (*p* = 0.015) (as presented in Table 4).

About the mean plaque scores in terms of gender disparity in study and control groups. The mean plaque scores before and after the interventions in females were as follows: group 1: 1.52 ± 0.20 and 0.36 ± 0.25, group 2: 1.38 ± 0.24 and 0.50 ± 0.37, and group 3: 1.58 ± 0.25 and 0.89 ± 0.23. Furthermore, for males, the mean plaque scores before and after the interventions were as follows: group 1: 1.54 ± 0.21 and 0.36 ± 0.29, group 2: 1.57 ± 0.26 and 0.69 ± 0.42, and group 3: 1.48 ± 0.23 and 0.96 ± 0.47. For females, a statistically significant difference was noted in mean plaque scores amongst the 3 groups after the provision of educational intervention (*p* = 0.004). Similarly, a statistically significant difference (*p* = 0.007) was found in mean plaque scores amongst the 3 groups for males. However, at preinterventional levels, no significant difference (*p* > 0.05) was found amongst all 3 groups in both sexes (as presented in Table 5).

About mean gingival scores in terms of gender between the groups, for females, the mean gingival scores before and after the interventions were as follows: group 1: 1.24 ± 0.12 and 0.34 ± 0.24, group 2: 1.31 ± 0.30 and 0.30 ± 0.39, and group 3: 1.52 ± 0.41 and 0.79 ± 0.54. For males, the mean gingival scores before and after the interventions were as follows: group 1: 1.34 ± 0.27 and 0.24 ± 0.22, group 2: 1.45 ± 0.29 and 0.56 ± 0.33, and group 3: 1.45 ± 0.23 and 0.80 ± 0.40. For females, no statistically significant difference was noted in mean gingival scores amongst the three groups (*p* = 0.095). However, a statistically significant difference was found in mean gingival scores amongst the 3 groups (*p* < 0.001) for males (as presented in Table 6).

Regarding OHI status of the 3 groups postintervention, “excellent” oral hygiene was noted in 4 (20%) hearing-impaired children in group 1, 2 (10%) in group 2, and none reported having “excellent” oral hygiene in group 3. “Good” oral hygiene was noted in 15 (75%) children belonging to group 1, 14 (70%) in group 2, and 10 (52.63%) in group 3, respectively. Moreover, 1 (5%) child in group 1, 4 (20%) in group 2, and 9 (47.36%) in group 3 were found to have “fair” oral hygiene.

About the health of the gingiva (GI), 5 hearing impaired children (25%) both in groups 1 and 2 reported having “healthy” gingiva along with 3 children (15.78%) from group 3. Furthermore, “mild” gingivitis was found in 15 (75%) children in group 1, 14 children (70%) in group 2, and 9 children (47.36%) in group 3.

Lastly, “moderate” gingivitis was not established in any of the children belonging to group 1, but it was found in 1 child (5%) in group 2, and 7 children (77.77%) in group 3 (as presented in Table 7).

## 4. Discussion

Maintaining good and healthy oral hygiene is of vital importance that plays a pivotal part in maintaining general physical health [16]. Normally, children are prone to have less than healthy oral health since at this age it is overlooked. Since children do not pay attention to their oral health, parents' guidance and education introduce many preventive dentistry methods such as tooth brushing and the use of dental floss [17]. However, for children with disabilities such as hearing impairment, difficulty in communication primarily hinders in maintaining healthy oral health most of the time [18].

In literature, many studies have evaluated different strategies to improve oral health such as giving direct or indirect personal tasks, written instructions, and audio-visual modalities provision [19]. In our study, the first intervention that we used was video as an oral hygiene education intervention, to help children with hearing impairment improve their oral health. According to our findings, video-based interventions significantly decreased both the mean plaque and gingival scores for the children as compared to no interventions being implemented. These findings correspond with a study by Widati and Nurmala, where a decrease in plaque scores was noted by using audio-visual aid and pamphlets [20]. Similarly, another study by Baliga et al. reports a decrease in plaque scores when oral health educational video was used as an oral hygiene education intervention [21]. The advantages associated with video-based interventions are that they can be repeatedly seen and mimicked by the children when they are brushing their teeth and using dental floss, along with being cost-effective.

The second intervention that was used in this study was the pictorial method as oral hygiene education to improve oral health in hearing impaired children. In this study, a significant reduction in mean plaque and gingival scores were noted when children were shown pictures of oral hygiene instructions. These findings correspond to various studies where improvement in oral health education was noted in hearing impaired children by showing pictures in a storybook [22, 23] Moreover, the use of sign language and lip-reading has also been used as a strategy in literature to guide hearing impaired children in managing their oral health [24]. A study in literature concludes a mean reduction in simplified oral hygiene index, gingival index, and plaque index by the use of sign language as a method of giving dental health education [25].

Tooth brushing can be performed using various techniques described in the literature such as horizontal scrub technique, bass techniques, and modified bass technique [26]. For children, horizontal scrub techniques have been recommended for the effective removal of plaque [27, 28]. Similarly, in our study, we recommended the children to use the horizontal scrub technique to brush their teeth. However, compliance to a particular tooth brushing technique can be a challenging factor to overcome especially for children. Toothbrushing along with mouthwash can be used to improve oral health according to one study where the use of mouthwash resulted in a decrease in gingival and plaque index scores [29].

It has been reported multiple times in the studies that females tend to have better oral hygiene as compared to males [30]. This might be due to females tend to visit dentists more often as compared to males and are more concerned about their oral hygiene [31]. In our study a mean reduction in plaque scores was reported in both males and females in postintervention; however, the reduction was slightly greater in females as compared to males. Furthermore, the reduction in the mean gingival scores of males was slightly more significant as compared to females. These results contrast with a study in literature where a significant difference between gingival scores of females and males was noted, with females having healthier gingiva [32].

Oral hygiene has been categorized as excellent, good, and fair in our study. In all of the 3 groups included in this study, the majority of the children belonging to group 1 (pictorial), and group 2 (video) had excellent and good oral hygiene at the end of the interventions assigned to each of the two groups. However, group 3 where no intervention was implemented mainly averaged fair in the oral hygiene status. Such results correspond to a study in literature where similar oral hygiene improvement has been found in intellectually disabled children by using oral hygiene educational interventions [33]. The use of electronic toothbrushes has been studied and proved effective for the better removal of plaque in children. However, a study showed no reduction in plaque when an electronic toothbrush was used in a child with special needs [34].

Oral hygiene educational interventions such as pictorial and video methods have been proven to be useful tools in improving the oral health of hearing-impaired children. However, other motivation techniques such as video games, sign language, and dental models have been used to improve the oral hygiene of hearing-impaired children [8, 35]. Despite the strengths of this study such as allocation of children to interventional groups randomly to avoid biases and following ADA (American Dental Association) and CONSORT guidelines, there were few limitations. Firstly, only a visual examination was used to assess the dental plaque without using plaque disclosing agents. Secondly, a few participants were recruited in this study, and lastly, only 2 motivational sessions were organized for the children.

Due to significant reduction of dental plaque in hearing-impaired children's students after oral hygiene education in this study. Properly planned educational programs are recommended to target oral health improvement to overcome the communication barriers. We also recommend a similar study on a large scale, with a longer duration greater than six months, maybe conducted in hearing impaired children. The participants may be exposed to the oral health educational interventions at least twice a week for three months to ensure the effectiveness of the educational interventions.

## 5. Conclusion

Oral hygiene is a vital aspect of the well-being of every individual due to functional and esthetic reasons. Children who have special needs such as being hearing impaired have difficulty in various aspects of life with oral health maintenance being one of them. Oral hygiene educational interventions such as the use of pictorial and video methods to educate hearing impaired children have been proven to be effective in improving the oral health of such children by a reduction in mean plaque and gingival scores.

## Figures and Tables

**Table 1:** Age and gender distribution of different groups (*n* = 59).

Demographics	Group 1	Group 2	Group 3
	*n* (%)	*n* (%)	*n* (%)
Age			
12-13 years	12 (60.0%)	12 (60.0%)	10 (52.6%)
14-16 years	8 (40.0%)	8 (40.0%)	9 (47.4%)
Gender			
Male	11 (55.0%)	11 (55.0%)	15 (78.9%)
Female	9 (45.0%)	9 (45.0%)	4 (21.1%)

**Table 2:** Comparison of mean plaque scores at pre- and postintervention levels in study groups.

Groups	Plaque scores		
	Preintervention	Postintervention	Pre-post
	Mean ± SD	Mean ± SD	Mean difference
Group 1 (pictorial)	1.53 ± 0.20	0.36 ± 0.26	1.17
Group 2 (video)	1.48 ± 0.26	0.60 ± 0.40	0.88
Group 3 (control)	1.50 ± 0.23	0.94 ± 0.43	0.56
*p* value	0.807	<0.001	—

SD: standard deviation.

**Table 3:** Comparison of mean gingival scores at pre- and postintervention levels in study groups.

Groups	Gingival scores		
	Preintervention	Postintervention	Pre-post
	Mean ± SD	Mean ± SD	Mean difference
Group 1 (pictorial)	1.29 ± 0.22	0.29 ± 0.23	1.00
Group 2 (video)	1.39 ± 0.30	0.44 ± 0.37	0.95
Group 3 (control)	1.47 ± 0.26	0.80 ± 0.42	0.67
*p* value	0.144	<0.001	—

SD: standard deviation.

**Table 4:** Intergroup OHI and GI scores comparison postinterventions.

Groups	OHI scores			GI scores		
	Mean difference	95% CI	*p* value	Mean difference	95% CI	*p* value
Group 1 (pictorial) vs. group 2 (video)	-0.097	-0.27, 0.07	0.537	-0.124	-0.31, 0.05	0.290
Group 1 (pictorial) vs. group 3 (control)	-0.274	-0.45, -0.09	0.001	-0.342	-0.52, -0.15	0.001
Group 2 (video) vs. group 3 (control)	-0.177	-0.35, 0.01	0.052	-0.218	-0.40, -0.03	0.015

OHI: oral hygiene index; GI: gingival index; CI: confidence interval; *p* value of ≤0.05 was considered as statistically significant.

**Table 5:** Comparison of mean plaque scores preintervention and postintervention in both sexes.

	Plaque scores		
	*n*	Preintervention	Postintervention
		Mean ± SD	Mean ± SD
*Gender*			
Females	22		
Group 1 (pictorial)	9	1.52 ± 0.20	0.36 ± 0.25
Group 2 (video)	9	1.38 ± 0.24	0.50 ± 0.37
Group 3 (control)	4	1.58 ± 0.25	0.89 ± 0.23
*p* value		0.608	0.004
Males	37		
Group 1 (pictorial)	11	1.54 ± 0.21	0.36 ± 0.29
Group 2 (video)	11	1.57 ± 0.26	0.69 ± 0.42
Group 3 (control)	15	1.48 ± 0.23	0.96 ± 0.47
*p* value		0.719	0.007

SD: standard deviation.

**Table 6:** Comparison of mean gingival score preintervention and postintervention in both sexes.

	Gingival scores		
	*n*	Preintervention	Postintervention
		Mean ± SD	Mean ± SD
*Gender*			
Females	22		
Group 1 (pictorial)	9	1.24 ± 0.12	0.34 ± 0.24
Group 2 (video)	9	1.31 ± 0.30	0.30 ± 0.39
Group 3 (control)	4	1.52 ± 0.41	0.79 ± 0.54
*p* value	—	0.242	0.095
Males	37		
Group 1 (pictorial)	11	1.34 ± 0.27	0.24 ± 0.22
Group 2 (video)	11	1.45 ± 0.29	0.56 ± 0.33
Group 3 (control)	15	1.45 ± 0.23	0.80 ± 0.40
*p* value	—	0.523	0.001

SD: standard deviation, *p* value of ≤0.05 was taken as significant.

**Table 7:** Distribution of oral hygiene index and gingival index amongst participants at postintervention levels in study groups.

	Group 1 (pictorial)	Group 2 (video)	Group 3 (control)
	*n* (%)	*n* (%)	*n* (%)
Post-OHI status			
Excellent	04 (20)	02 (10)	00 (0)
Good	15 (75)	14 (70)	10 (52.63)
Fair	01 (5)	04 (20)	09 (47.36)
Post-GI status			
Healthy	05 (25)	05 (25)	03 (15.78)
Mild	15 (75)	14 (70)	09 (47.36)
Moderate	0 (0)	01 (5)	07 (77.77)

OHI: oral hygiene index; GI: gingival index; %: percentage; *n*: frequency.

## Data Availability

The raw data used to support the findings of this study are included within the article.
